# 3-{4-[(2-Hy­droxy­benzyl­idene)amino]-3-methyl-5-sulfanyl­idene-4,5-dihydro-1*H*-1,2,4-triazol-1-yl}-1,3-diphenyl­propan-1-one

**DOI:** 10.1107/S1600536811034878

**Published:** 2011-08-31

**Authors:** Wei Wang, Yan Gao, Jing-jing Zhang, Xiao-yu Jia, Wen-peng Wu

**Affiliations:** a School of Perfume and Aroma Technology, Shanghai Institute of Technology, Shanghai 200235, People’s Republic of China; b School of Chemical Engineering, University of Science and Technology LiaoNing, Anshan 114051, People’s Republic of China

## Abstract

There are two crystallographically independent mol­ecules (*A* and *B*) in the asymmetric unit of the title compound, C_25_H_22_N_4_O_2_S, with almost identical mol­ecular conformations. The hy­droxy­phenyl ring plane and the 1,2,4-triazole ring form dihedral angles of 17.1 (2) and 7.4 (2)° in *A* and *B*, respectively. The dihedral angles between 1,2,4-triazole ring and the other two phenyl rings are 89.6 (3) and 83.3 (2)° in mol­ecule *A*, and 89.2 (3) and 82.2 (2)° in mol­ecule *B*. One intra­molecular O—H⋯N hydrogen bond is present in each mol­ecule. Weak inter­molecular C—H⋯O hydrogen bonds consolidate the crystal packing.

## Related literature

For the crystal structures of related 1,2,4-triazole-5(4*H*)-thione derivatives, see: Al-Tamimi *et al.* (2010 ▶); Fun *et al.* (2009 ▶); Gao *et al.* (2011 ▶); Tan *et al.* (2010 ▶); Wang *et al.* (2011 ▶); Zhao *et al.* (2010 ▶).
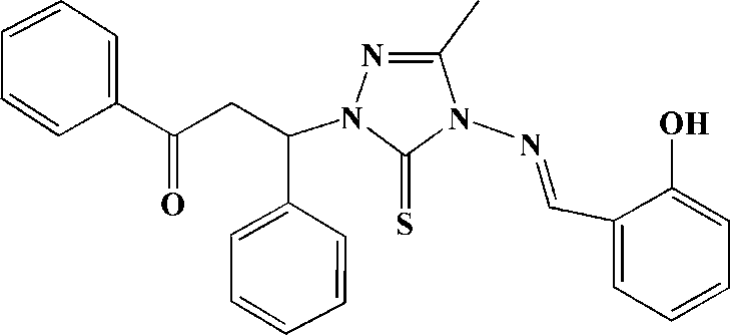


## Experimental

### 

#### Crystal data

C_25_H_22_N_4_O_2_S
*M* *_r_* = 442.53
Triclinic, 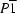

*a* = 12.1053 (12) Å
*b* = 12.4682 (12) Å
*c* = 15.5480 (16) Å
α = 95.056 (11)°
β = 103.342 (12)°
γ = 100.212 (15)°
*V* = 2226.7 (4) Å^3^
*Z* = 4
Mo *K*α radiation
μ = 0.18 mm^−1^
*T* = 113 K
0.20 × 0.18 × 0.12 mm

#### Data collection

Rigaku Saturn CCD area-detector diffractometer
Absorption correction: multi-scan (*CrystalClear*; Rigaku/MSC, 2005 ▶) *T*_min_ = 0.966, *T*_max_ = 0.979
27078 measured reflections
9643 independent reflections
6461 reflections with *I* > 2σ(*I*)
*R*_int_ = 0.073

#### Refinement

*R*[*F* ^2^ > 2σ(*F* ^2^)] = 0.064
*wR*(*F* ^2^) = 0.145
*S* = 1.00
9643 reflections
585 parameters
H atoms treated by a mixture of independent and constrained refinement
Δρ_max_ = 0.61 e Å^−3^
Δρ_min_ = −0.33 e Å^−3^

### 

Data collection: *CrystalClear* (Rigaku/MSC, 2005 ▶); cell refinement: *CrystalClear*; data reduction: *CrystalClear*; program(s) used to solve structure: *SHELXS97* (Sheldrick, 2008 ▶); program(s) used to refine structure: *SHELXL97* (Sheldrick, 2008 ▶); molecular graphics: *SHELXTL* (Sheldrick, 2008 ▶); software used to prepare material for publication: *SHELXTL*.

## Supplementary Material

Crystal structure: contains datablock(s) global, I. DOI: 10.1107/S1600536811034878/bh2375sup1.cif

Structure factors: contains datablock(s) I. DOI: 10.1107/S1600536811034878/bh2375Isup2.hkl

Supplementary material file. DOI: 10.1107/S1600536811034878/bh2375Isup3.cml

Additional supplementary materials:  crystallographic information; 3D view; checkCIF report

## Figures and Tables

**Table 1:** Hydrogen-bond geometry (Å, °)

*D*—H⋯*A*	*D*—H	H⋯*A*	*D*⋯*A*	*D*—H⋯*A*
O2—H2⋯N4	0.85 (3)	1.92 (3)	2.653 (3)	144 (3)
O4—H4⋯N8	0.95 (3)	1.80 (3)	2.658 (3)	149 (3)
C29—H29*B*⋯O1^i^	0.99	2.52	3.331 (3)	139 (2)
C38—H38⋯O1^i^	0.95	2.56	3.440 (3)	154 (2)

Symmetry code: (i) 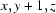.

**Fig. 1.**
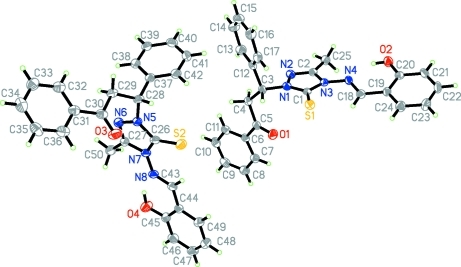
View of the asymmetric unit for the title molecule, with displacement ellipsoids drawn at the 60% probability level.

## supplementary crystallographic information

### Comment

In continuation of structural study of Mannich bases derivatives synthesized by reactions of the amino heterocycles and aromatic aldehydes in our group (Wang *et al.*, 2011), we present here the crystal structure of the title compound.

There are two independent molecules (*A* and *B*) in the asymmetric unit (Fig. 1). The two molecules have slightly different bond lengths and the bond lengths and angles in the compound are found to have normal values, compared to those reported in the related 1,2,4-triazole-5(4*H*)-thione derivatives (Al-Tamimi *et al.*, 2010; Fun *et al.*, 2009; Tan *et al.*, 2010; Wang *et al.*, 2011). The two independent molecular conformations are similar, the hydroxyphenyl ring plane and 1,2,4-triazole ring forming dihedral angles of 17.1 (2) and 7.4 (2)°, in *A* and *B*, respectively. The dihedral angles between 1,2,4-triazole ring and the other two phenyl rings are 89.6 (3) and 83.3 (2)°, respectively in molecule *A*, and 89.2 (3) and 82.2 (2)° in molecule *B*. Two C atoms in the 1,2,4-triazole ring show distorted C*sp*^2^ hybridization states with the bond angles of 101.2 (2)° (N1—C1—N3), 130.68 (19)° (N3—C1—S1), 110.8 (2)° (N2—C2—N3) and 125.7 (2)° (N2—C2—C25) in molecule *A* [the corresponding values are 101.8 (2), 131.0 (2), 111.2 (2) and 125.1 (2)° in molecule *B*], which are similar to the reported triazole derivatives (Zhao *et al.*, 2010; Gao *et al.*, 2011).

An intramolecular O—H···N hydrogen bond is present in each molecule. The crystal structure is consolidated by weak intermolecular C—H···O hydrogen bonds (Table 1).

### Experimental

The title compound was synthesized by the reaction of 2-hydroxybenzaldehyde (2.0 mmol) and 3-(4-amino-3-methyl-5-thioxo-4,5-dihydro-1*H*-1,2,4-triazol-1-yl)-1,3-diphenylpropan-1-one (2.0 mmol) in refluxing ethanol. The reaction progress was monitored *via* TLC. The resulting precipitate was filtered off, washed with cold ethanol, dried and purified to give the target product as a colorless solid in 73% yield. Crystals suitable for single-crystal X-ray analysis were grown by slow evaporation of a solution in chloroform-ethanol (1:1).

### Refinement

The H atoms attached to O atoms were located in a different density map and the atomic coordinations allowed to refine freely. Other H atoms were positioned geometrically and refined as riding (C—H = 0.95–1.00 Å) and allowed to ride on their parent atoms, with *U*_iso_(H) = 1.2*U*_eq_(parent) or 1.5*U*_eq_(parent).

### Crystal data

**Table:**

C_25_H_22_N_4_O_2_S	*Z* = 4
*M**_r_* = 442.53	*F*(000) = 928
Triclinic, *P*1	*D*_x_ = 1.320 Mg m^−^^3^
Hall symbol: -P 1	Mo *K*α radiation, λ = 0.71073 Å
*a* = 12.1053 (12) Å	Cell parameters from 6881 reflections
*b* = 12.4682 (12) Å	θ = 1.4–27.1°
*c* = 15.5480 (16) Å	µ = 0.18 mm^−^^1^
α = 95.056 (11)°	*T* = 113 K
β = 103.342 (12)°	Prism, colourless
γ = 100.212 (15)°	0.20 × 0.18 × 0.12 mm
*V* = 2226.7 (4) Å^3^	

### Data collection

**Table:**

Rigaku Saturn CCD area-detector diffractometer	9643 independent reflections
Radiation source: rotating anode	6461 reflections with *I* > 2σ(*I*)
multilayer	*R*_int_ = 0.073
Detector resolution: 14.63 pixels mm^-1^	θ_max_ = 27.0°, θ_min_ = 1.4°
φ and ω scans	*h* = −15→14
Absorption correction: multi-scan (*CrystalClear*; Rigaku/MSC, 2005)	*k* = −15→15
*T*_min_ = 0.966, *T*_max_ = 0.979	*l* = −19→19
27078 measured reflections	

### Refinement

**Table:**

Refinement on *F*^2^	Primary atom site location: structure-invariant direct methods
Least-squares matrix: full	Secondary atom site location: difference Fourier map
*R*[*F*^2^ > 2σ(*F*^2^)] = 0.064	Hydrogen site location: inferred from neighbouring sites
*wR*(*F*^2^) = 0.145	H atoms treated by a mixture of independent and constrained refinement
*S* = 1.00	*w* = 1/[σ^2^(*F*_o_^2^) + (0.056*P*)^2^] where *P* = (*F*_o_^2^ + 2*F*_c_^2^)/3
9643 reflections	(Δ/σ)_max_ < 0.001
585 parameters	Δρ_max_ = 0.61 e Å^−^^3^
0 restraints	Δρ_min_ = −0.33 e Å^−^^3^
0 constraints	

### Fractional atomic coordinates and isotropic or equivalent isotropic displacement parameters (Å^2^)

**Table:**

	*x*	*y*	*z*	*U*_iso_*/*U*_eq_	
S1	0.92386 (6)	0.21027 (5)	0.23449 (5)	0.02221 (18)	
S2	0.83187 (6)	0.71656 (6)	0.49822 (5)	0.02859 (19)	
O1	0.62968 (15)	0.17443 (15)	0.27421 (13)	0.0274 (5)	
O2	0.90261 (17)	−0.16179 (16)	−0.04063 (13)	0.0295 (5)	
H2	0.866 (3)	−0.113 (2)	−0.027 (2)	0.044*	
O3	0.88771 (17)	1.03662 (15)	0.56160 (13)	0.0332 (5)	
O4	0.38942 (16)	0.58080 (16)	0.60955 (14)	0.0330 (5)	
H4	0.431 (3)	0.627 (2)	0.577 (2)	0.049*	
N1	0.71659 (18)	0.20564 (16)	0.11772 (14)	0.0183 (5)	
N2	0.64145 (18)	0.15040 (17)	0.03849 (15)	0.0204 (5)	
N3	0.79130 (17)	0.07125 (16)	0.08120 (14)	0.0172 (5)	
N4	0.85762 (18)	−0.00627 (16)	0.06624 (15)	0.0199 (5)	
N5	0.70663 (18)	0.86606 (17)	0.43391 (15)	0.0205 (5)	
N6	0.59842 (18)	0.89447 (17)	0.42255 (15)	0.0224 (5)	
N7	0.60683 (18)	0.74759 (17)	0.49396 (14)	0.0199 (5)	
N8	0.56141 (18)	0.66959 (17)	0.54196 (14)	0.0210 (5)	
C1	0.8117 (2)	0.1620 (2)	0.14674 (17)	0.0168 (6)	
C2	0.6902 (2)	0.0697 (2)	0.01751 (17)	0.0184 (6)	
C3	0.6948 (2)	0.31047 (19)	0.15598 (18)	0.0186 (6)	
H3	0.7493	0.3323	0.2165	0.022*	
C4	0.5720 (2)	0.2947 (2)	0.16793 (18)	0.0210 (6)	
H4A	0.5165	0.2660	0.1095	0.025*	
H4B	0.5570	0.3670	0.1884	0.025*	
C5	0.5508 (2)	0.2159 (2)	0.23477 (18)	0.0206 (6)	
C6	0.4334 (2)	0.1894 (2)	0.25256 (17)	0.0193 (6)	
C7	0.4119 (2)	0.1069 (2)	0.30634 (18)	0.0238 (6)	
H7	0.4700	0.0668	0.3277	0.029*	
C8	0.3057 (2)	0.0837 (2)	0.32840 (19)	0.0272 (7)	
H8	0.2918	0.0283	0.3651	0.033*	
C9	0.2200 (2)	0.1419 (2)	0.29662 (19)	0.0280 (7)	
H9	0.1484	0.1274	0.3130	0.034*	
C10	0.2389 (2)	0.2212 (2)	0.24093 (19)	0.0263 (7)	
H10	0.1794	0.2593	0.2180	0.032*	
C11	0.3454 (2)	0.2448 (2)	0.21884 (18)	0.0232 (6)	
H11	0.3581	0.2987	0.1807	0.028*	
C12	0.7260 (2)	0.3993 (2)	0.09762 (18)	0.0218 (6)	
C13	0.6453 (2)	0.4232 (2)	0.02656 (19)	0.0280 (7)	
H13	0.5661	0.3882	0.0156	0.034*	
C14	0.6790 (3)	0.4981 (2)	−0.0292 (2)	0.0336 (7)	
H14	0.6228	0.5136	−0.0777	0.040*	
C15	0.7950 (3)	0.5500 (2)	−0.0134 (2)	0.0336 (7)	
H15	0.8181	0.6013	−0.0508	0.040*	
C16	0.8761 (3)	0.5263 (2)	0.0568 (2)	0.0326 (7)	
H16	0.9554	0.5610	0.0673	0.039*	
C17	0.8422 (2)	0.4514 (2)	0.1126 (2)	0.0267 (7)	
H17	0.8985	0.4359	0.1610	0.032*	
C18	0.9372 (2)	−0.0246 (2)	0.13119 (18)	0.0218 (6)	
H18	0.9503	0.0139	0.1893	0.026*	
C19	1.0066 (2)	−0.1046 (2)	0.11477 (18)	0.0190 (6)	
C20	0.9894 (2)	−0.1674 (2)	0.03076 (18)	0.0208 (6)	
C21	1.0628 (2)	−0.2392 (2)	0.0193 (2)	0.0262 (7)	
H21	1.0514	−0.2812	−0.0372	0.031*	
C22	1.1522 (2)	−0.2495 (2)	0.0898 (2)	0.0285 (7)	
H22	1.2027	−0.2974	0.0809	0.034*	
C23	1.1690 (2)	−0.1903 (2)	0.1739 (2)	0.0312 (7)	
H23	1.2291	−0.1990	0.2226	0.037*	
C24	1.0965 (2)	−0.1186 (2)	0.1852 (2)	0.0278 (7)	
H24	1.1080	−0.0778	0.2423	0.033*	
C25	0.6467 (2)	−0.0121 (2)	−0.06449 (18)	0.0248 (6)	
H25A	0.5715	−0.0007	−0.0980	0.037*	
H25B	0.6378	−0.0865	−0.0478	0.037*	
H25C	0.7021	−0.0028	−0.1017	0.037*	
C26	0.7169 (2)	0.7757 (2)	0.47639 (17)	0.0209 (6)	
C27	0.5405 (2)	0.8219 (2)	0.46061 (18)	0.0212 (6)	
C28	0.7919 (2)	0.9226 (2)	0.38905 (18)	0.0221 (6)	
H28	0.8686	0.9038	0.4151	0.027*	
C29	0.8065 (2)	1.0476 (2)	0.40721 (18)	0.0235 (6)	
H29A	0.8567	1.0820	0.3709	0.028*	
H29B	0.7297	1.0670	0.3874	0.028*	
C30	0.8588 (2)	1.0960 (2)	0.50451 (19)	0.0233 (6)	
C31	0.8727 (2)	1.2177 (2)	0.52909 (19)	0.0227 (6)	
C32	0.8483 (2)	1.2898 (2)	0.4661 (2)	0.0270 (7)	
H32	0.8196	1.2617	0.4046	0.032*	
C33	0.8659 (2)	1.4022 (2)	0.4933 (2)	0.0312 (7)	
H33	0.8492	1.4504	0.4502	0.037*	
C34	0.9079 (2)	1.4445 (2)	0.5830 (2)	0.0338 (8)	
H34	0.9210	1.5214	0.6011	0.041*	
C35	0.9306 (3)	1.3733 (2)	0.6466 (2)	0.0357 (8)	
H35	0.9579	1.4016	0.7081	0.043*	
C36	0.9132 (2)	1.2612 (2)	0.6194 (2)	0.0291 (7)	
H36	0.9290	1.2132	0.6628	0.035*	
C37	0.7548 (2)	0.8752 (2)	0.29097 (19)	0.0214 (6)	
C38	0.6689 (2)	0.9106 (2)	0.22952 (19)	0.0240 (6)	
H38	0.6368	0.9703	0.2478	0.029*	
C39	0.6302 (2)	0.8584 (2)	0.14137 (19)	0.0252 (6)	
H39	0.5709	0.8818	0.1004	0.030*	
C40	0.6784 (2)	0.7724 (2)	0.1137 (2)	0.0281 (7)	
H40	0.6528	0.7376	0.0537	0.034*	
C41	0.7640 (3)	0.7373 (2)	0.1738 (2)	0.0303 (7)	
H41	0.7972	0.6787	0.1550	0.036*	
C42	0.8011 (2)	0.7882 (2)	0.2617 (2)	0.0270 (7)	
H42	0.8592	0.7631	0.3025	0.032*	
C43	0.6233 (2)	0.6041 (2)	0.58012 (17)	0.0222 (6)	
H43	0.6998	0.6069	0.5734	0.027*	
C44	0.5754 (2)	0.5258 (2)	0.63360 (18)	0.0210 (6)	
C45	0.4626 (2)	0.5166 (2)	0.64556 (18)	0.0237 (6)	
C46	0.4213 (3)	0.4391 (2)	0.69749 (19)	0.0293 (7)	
H46	0.3445	0.4321	0.7045	0.035*	
C47	0.4928 (3)	0.3731 (2)	0.7383 (2)	0.0320 (7)	
H47	0.4647	0.3208	0.7732	0.038*	
C48	0.6053 (3)	0.3830 (2)	0.7286 (2)	0.0338 (7)	
H48	0.6545	0.3385	0.7576	0.041*	
C49	0.6457 (3)	0.4583 (2)	0.67630 (19)	0.0295 (7)	
H49	0.7224	0.4641	0.6694	0.035*	
C50	0.4191 (2)	0.8177 (2)	0.46712 (19)	0.0274 (7)	
H50A	0.3895	0.8777	0.4390	0.041*	
H50B	0.4173	0.8256	0.5300	0.041*	
H50C	0.3705	0.7471	0.4366	0.041*	

### Atomic displacement parameters (Å^2^)

**Table:**

	*U*^11^	*U*^22^	*U*^33^	*U*^12^	*U*^13^	*U*^23^
S1	0.0204 (4)	0.0242 (4)	0.0210 (4)	0.0043 (3)	0.0043 (3)	0.0010 (3)
S2	0.0263 (4)	0.0337 (4)	0.0344 (5)	0.0131 (3)	0.0154 (3)	0.0159 (3)
O1	0.0244 (11)	0.0308 (11)	0.0309 (12)	0.0108 (9)	0.0087 (9)	0.0094 (9)
O2	0.0351 (12)	0.0342 (12)	0.0209 (11)	0.0161 (10)	0.0049 (10)	−0.0002 (9)
O3	0.0411 (13)	0.0337 (12)	0.0240 (12)	0.0061 (10)	0.0050 (10)	0.0113 (9)
O4	0.0257 (12)	0.0370 (13)	0.0420 (14)	0.0079 (9)	0.0135 (10)	0.0189 (10)
N1	0.0186 (12)	0.0173 (11)	0.0190 (12)	0.0014 (9)	0.0063 (10)	0.0022 (9)
N2	0.0188 (12)	0.0212 (12)	0.0207 (13)	0.0022 (10)	0.0060 (10)	0.0019 (10)
N3	0.0181 (12)	0.0172 (11)	0.0178 (12)	0.0039 (9)	0.0067 (10)	0.0044 (9)
N4	0.0207 (12)	0.0184 (12)	0.0249 (13)	0.0068 (9)	0.0116 (10)	0.0051 (10)
N5	0.0200 (12)	0.0235 (12)	0.0219 (13)	0.0069 (10)	0.0097 (10)	0.0072 (10)
N6	0.0211 (12)	0.0256 (13)	0.0232 (13)	0.0072 (10)	0.0080 (10)	0.0061 (10)
N7	0.0213 (12)	0.0222 (12)	0.0182 (12)	0.0043 (10)	0.0078 (10)	0.0061 (10)
N8	0.0257 (13)	0.0196 (12)	0.0188 (12)	0.0016 (10)	0.0089 (10)	0.0054 (9)
C1	0.0183 (14)	0.0163 (13)	0.0189 (14)	0.0020 (11)	0.0104 (11)	0.0067 (11)
C2	0.0200 (14)	0.0168 (14)	0.0192 (14)	0.0023 (11)	0.0070 (12)	0.0045 (11)
C3	0.0209 (14)	0.0174 (14)	0.0178 (14)	0.0052 (11)	0.0052 (12)	−0.0005 (11)
C4	0.0209 (15)	0.0220 (14)	0.0229 (15)	0.0079 (11)	0.0080 (12)	0.0038 (12)
C5	0.0239 (15)	0.0205 (14)	0.0174 (14)	0.0068 (12)	0.0047 (12)	−0.0007 (11)
C6	0.0191 (14)	0.0234 (15)	0.0146 (14)	0.0049 (11)	0.0033 (11)	−0.0008 (11)
C7	0.0256 (15)	0.0230 (15)	0.0209 (15)	0.0026 (12)	0.0050 (12)	0.0000 (12)
C8	0.0266 (16)	0.0284 (16)	0.0267 (17)	0.0016 (13)	0.0100 (13)	0.0034 (13)
C9	0.0216 (16)	0.0328 (17)	0.0277 (17)	0.0003 (13)	0.0090 (13)	−0.0021 (13)
C10	0.0209 (15)	0.0314 (16)	0.0254 (16)	0.0069 (12)	0.0043 (13)	−0.0025 (13)
C11	0.0228 (15)	0.0270 (15)	0.0193 (15)	0.0049 (12)	0.0053 (12)	0.0001 (12)
C12	0.0261 (16)	0.0215 (15)	0.0214 (15)	0.0085 (12)	0.0110 (13)	0.0007 (11)
C13	0.0275 (16)	0.0283 (16)	0.0280 (17)	0.0035 (13)	0.0083 (14)	0.0040 (13)
C14	0.046 (2)	0.0318 (17)	0.0267 (17)	0.0108 (15)	0.0119 (15)	0.0085 (13)
C15	0.049 (2)	0.0248 (16)	0.0341 (19)	0.0060 (14)	0.0235 (16)	0.0078 (14)
C16	0.0323 (18)	0.0248 (16)	0.046 (2)	0.0042 (13)	0.0200 (16)	0.0066 (14)
C17	0.0256 (16)	0.0222 (15)	0.0343 (18)	0.0072 (12)	0.0103 (14)	0.0025 (13)
C18	0.0241 (15)	0.0206 (14)	0.0207 (15)	0.0044 (12)	0.0063 (12)	0.0025 (11)
C19	0.0187 (14)	0.0164 (14)	0.0235 (15)	0.0037 (11)	0.0074 (12)	0.0049 (11)
C20	0.0229 (15)	0.0201 (14)	0.0222 (15)	0.0036 (11)	0.0101 (13)	0.0064 (11)
C21	0.0317 (17)	0.0232 (15)	0.0305 (17)	0.0083 (13)	0.0187 (14)	0.0046 (13)
C22	0.0265 (16)	0.0250 (16)	0.042 (2)	0.0136 (13)	0.0174 (15)	0.0092 (14)
C23	0.0279 (17)	0.0317 (17)	0.0346 (19)	0.0129 (14)	0.0032 (14)	0.0067 (14)
C24	0.0296 (16)	0.0266 (16)	0.0262 (17)	0.0079 (13)	0.0042 (13)	0.0014 (13)
C25	0.0279 (16)	0.0233 (15)	0.0219 (16)	0.0055 (12)	0.0045 (13)	−0.0005 (12)
C26	0.0247 (15)	0.0213 (14)	0.0172 (14)	0.0022 (12)	0.0073 (12)	0.0048 (11)
C27	0.0225 (15)	0.0210 (14)	0.0223 (15)	0.0066 (12)	0.0078 (12)	0.0050 (11)
C28	0.0210 (15)	0.0264 (15)	0.0236 (16)	0.0063 (12)	0.0104 (12)	0.0121 (12)
C29	0.0241 (15)	0.0266 (15)	0.0211 (15)	0.0047 (12)	0.0066 (12)	0.0086 (12)
C30	0.0179 (15)	0.0323 (16)	0.0212 (16)	0.0044 (12)	0.0073 (12)	0.0065 (12)
C31	0.0153 (14)	0.0309 (16)	0.0228 (15)	0.0040 (12)	0.0064 (12)	0.0057 (12)
C32	0.0217 (15)	0.0334 (17)	0.0259 (16)	0.0043 (13)	0.0062 (13)	0.0066 (13)
C33	0.0293 (17)	0.0284 (17)	0.0357 (19)	0.0031 (13)	0.0087 (14)	0.0075 (14)
C34	0.0279 (17)	0.0307 (17)	0.041 (2)	0.0015 (13)	0.0121 (15)	−0.0028 (15)
C35	0.0364 (19)	0.0402 (19)	0.0274 (18)	0.0068 (15)	0.0068 (15)	−0.0064 (14)
C36	0.0230 (16)	0.0397 (18)	0.0257 (17)	0.0080 (13)	0.0060 (13)	0.0081 (14)
C37	0.0201 (14)	0.0231 (15)	0.0258 (16)	0.0050 (11)	0.0123 (12)	0.0098 (12)
C38	0.0256 (16)	0.0238 (15)	0.0269 (16)	0.0082 (12)	0.0116 (13)	0.0074 (12)
C39	0.0276 (16)	0.0256 (16)	0.0248 (16)	0.0060 (13)	0.0087 (13)	0.0091 (12)
C40	0.0341 (17)	0.0277 (16)	0.0248 (16)	0.0027 (13)	0.0141 (14)	0.0052 (13)
C41	0.0391 (18)	0.0258 (16)	0.0350 (19)	0.0131 (14)	0.0209 (15)	0.0081 (14)
C42	0.0267 (16)	0.0318 (17)	0.0294 (17)	0.0117 (13)	0.0134 (14)	0.0123 (13)
C43	0.0242 (15)	0.0247 (15)	0.0190 (15)	0.0047 (12)	0.0084 (12)	0.0029 (12)
C44	0.0269 (15)	0.0177 (14)	0.0190 (15)	0.0034 (11)	0.0073 (12)	0.0027 (11)
C45	0.0264 (16)	0.0205 (15)	0.0228 (16)	0.0036 (12)	0.0051 (13)	0.0016 (12)
C46	0.0346 (17)	0.0249 (16)	0.0292 (17)	0.0009 (13)	0.0135 (14)	0.0043 (13)
C47	0.048 (2)	0.0244 (16)	0.0278 (17)	0.0034 (14)	0.0186 (15)	0.0094 (13)
C48	0.045 (2)	0.0288 (17)	0.0345 (19)	0.0142 (14)	0.0149 (16)	0.0134 (14)
C49	0.0351 (18)	0.0310 (17)	0.0286 (17)	0.0116 (14)	0.0142 (14)	0.0112 (13)
C50	0.0252 (16)	0.0321 (17)	0.0289 (17)	0.0096 (13)	0.0097 (13)	0.0102 (13)

### Geometric parameters (Å, °)

**Table:**

S1—C1	1.667 (3)	C19—C20	1.414 (4)
S2—C26	1.669 (3)	C20—C21	1.397 (3)
O1—C5	1.231 (3)	C21—C22	1.384 (4)
O2—C20	1.358 (3)	C21—H21	0.9500
O2—H2	0.85 (3)	C22—C23	1.397 (4)
O3—C30	1.234 (3)	C22—H22	0.9500
O4—C45	1.356 (3)	C23—C24	1.387 (4)
O4—H4	0.95 (3)	C23—H23	0.9500
N1—C1	1.361 (3)	C24—H24	0.9500
N1—N2	1.389 (3)	C25—H25A	0.9800
N1—C3	1.479 (3)	C25—H25B	0.9800
N2—C2	1.307 (3)	C25—H25C	0.9800
N3—C2	1.381 (3)	C27—C50	1.488 (3)
N3—N4	1.398 (3)	C28—C37	1.519 (4)
N3—C1	1.400 (3)	C28—C29	1.531 (4)
N4—C18	1.292 (3)	C28—H28	1.0000
N5—C26	1.366 (3)	C29—C30	1.522 (4)
N5—N6	1.393 (3)	C29—H29A	0.9900
N5—C28	1.487 (3)	C29—H29B	0.9900
N6—C27	1.312 (3)	C30—C31	1.501 (4)
N7—C27	1.388 (3)	C31—C36	1.400 (4)
N7—N8	1.390 (3)	C31—C32	1.404 (4)
N7—C26	1.411 (3)	C32—C33	1.391 (4)
N8—C43	1.295 (3)	C32—H32	0.9500
C2—C25	1.487 (3)	C33—C34	1.390 (4)
C3—C4	1.521 (3)	C33—H33	0.9500
C3—C12	1.542 (3)	C34—C35	1.398 (4)
C3—H3	1.0000	C34—H34	0.9500
C4—C5	1.524 (4)	C35—C36	1.388 (4)
C4—H4A	0.9900	C35—H35	0.9500
C4—H4B	0.9900	C36—H36	0.9500
C5—C6	1.496 (4)	C37—C42	1.394 (4)
C6—C11	1.397 (3)	C37—C38	1.403 (4)
C6—C7	1.408 (4)	C38—C39	1.400 (4)
C7—C8	1.393 (4)	C38—H38	0.9500
C7—H7	0.9500	C39—C40	1.391 (4)
C8—C9	1.393 (4)	C39—H39	0.9500
C8—H8	0.9500	C40—C41	1.387 (4)
C9—C10	1.394 (4)	C40—H40	0.9500
C9—H9	0.9500	C41—C42	1.390 (4)
C10—C11	1.398 (4)	C41—H41	0.9500
C10—H10	0.9500	C42—H42	0.9500
C11—H11	0.9500	C43—C44	1.465 (3)
C12—C13	1.388 (4)	C43—H43	0.9500
C12—C17	1.397 (4)	C44—C49	1.401 (3)
C13—C14	1.401 (4)	C44—C45	1.407 (4)
C13—H13	0.9500	C45—C46	1.410 (4)
C14—C15	1.393 (4)	C46—C47	1.385 (4)
C14—H14	0.9500	C46—H46	0.9500
C15—C16	1.381 (4)	C47—C48	1.391 (4)
C15—H15	0.9500	C47—H47	0.9500
C16—C17	1.403 (4)	C48—C49	1.392 (4)
C16—H16	0.9500	C48—H48	0.9500
C17—H17	0.9500	C49—H49	0.9500
C18—C19	1.454 (3)	C50—H50A	0.9800
C18—H18	0.9500	C50—H50B	0.9800
C19—C24	1.402 (4)	C50—H50C	0.9800
			
C20—O2—H2	110 (2)	C23—C24—C19	121.8 (3)
C45—O4—H4	105.7 (18)	C23—C24—H24	119.1
C1—N1—N2	114.5 (2)	C19—C24—H24	119.1
C1—N1—C3	126.3 (2)	C2—C25—H25A	109.5
N2—N1—C3	118.7 (2)	C2—C25—H25B	109.5
C2—N2—N1	104.0 (2)	H25A—C25—H25B	109.5
C2—N3—N4	118.0 (2)	C2—C25—H25C	109.5
C2—N3—C1	109.41 (19)	H25A—C25—H25C	109.5
N4—N3—C1	132.3 (2)	H25B—C25—H25C	109.5
C18—N4—N3	120.0 (2)	N5—C26—N7	101.8 (2)
C26—N5—N6	114.3 (2)	N5—C26—S2	127.2 (2)
C26—N5—C28	125.7 (2)	N7—C26—S2	131.0 (2)
N6—N5—C28	119.4 (2)	N6—C27—N7	111.2 (2)
C27—N6—N5	104.1 (2)	N6—C27—C50	125.1 (2)
C27—N7—N8	118.7 (2)	N7—C27—C50	123.7 (2)
C27—N7—C26	108.6 (2)	N5—C28—C37	108.0 (2)
N8—N7—C26	132.4 (2)	N5—C28—C29	111.0 (2)
C43—N8—N7	120.9 (2)	C37—C28—C29	114.8 (2)
N1—C1—N3	101.2 (2)	N5—C28—H28	107.6
N1—C1—S1	128.02 (19)	C37—C28—H28	107.6
N3—C1—S1	130.68 (19)	C29—C28—H28	107.6
N2—C2—N3	110.8 (2)	C30—C29—C28	114.4 (2)
N2—C2—C25	125.7 (2)	C30—C29—H29A	108.7
N3—C2—C25	123.5 (2)	C28—C29—H29A	108.7
N1—C3—C4	110.4 (2)	C30—C29—H29B	108.7
N1—C3—C12	108.2 (2)	C28—C29—H29B	108.7
C4—C3—C12	115.3 (2)	H29A—C29—H29B	107.6
N1—C3—H3	107.5	O3—C30—C31	121.1 (3)
C4—C3—H3	107.5	O3—C30—C29	120.8 (2)
C12—C3—H3	107.5	C31—C30—C29	118.1 (2)
C3—C4—C5	112.9 (2)	C36—C31—C32	118.6 (3)
C3—C4—H4A	109.0	C36—C31—C30	118.1 (3)
C5—C4—H4A	109.0	C32—C31—C30	123.3 (3)
C3—C4—H4B	109.0	C33—C32—C31	120.3 (3)
C5—C4—H4B	109.0	C33—C32—H32	119.8
H4A—C4—H4B	107.8	C31—C32—H32	119.8
O1—C5—C6	120.0 (2)	C34—C33—C32	120.5 (3)
O1—C5—C4	120.4 (2)	C34—C33—H33	119.8
C6—C5—C4	119.6 (2)	C32—C33—H33	119.8
C11—C6—C7	119.1 (2)	C33—C34—C35	119.8 (3)
C11—C6—C5	122.3 (2)	C33—C34—H34	120.1
C7—C6—C5	118.6 (2)	C35—C34—H34	120.1
C8—C7—C6	120.4 (3)	C36—C35—C34	119.7 (3)
C8—C7—H7	119.8	C36—C35—H35	120.1
C6—C7—H7	119.8	C34—C35—H35	120.1
C9—C8—C7	119.9 (3)	C35—C36—C31	121.1 (3)
C9—C8—H8	120.0	C35—C36—H36	119.4
C7—C8—H8	120.0	C31—C36—H36	119.4
C8—C9—C10	120.2 (3)	C42—C37—C38	118.4 (3)
C8—C9—H9	119.9	C42—C37—C28	119.3 (2)
C10—C9—H9	119.9	C38—C37—C28	122.1 (2)
C9—C10—C11	119.9 (3)	C39—C38—C37	120.4 (3)
C9—C10—H10	120.0	C39—C38—H38	119.8
C11—C10—H10	120.0	C37—C38—H38	119.8
C6—C11—C10	120.3 (3)	C40—C39—C38	120.1 (3)
C6—C11—H11	119.8	C40—C39—H39	120.0
C10—C11—H11	119.8	C38—C39—H39	120.0
C13—C12—C17	118.7 (3)	C41—C40—C39	119.9 (3)
C13—C12—C3	122.8 (2)	C41—C40—H40	120.0
C17—C12—C3	118.3 (2)	C39—C40—H40	120.0
C12—C13—C14	121.0 (3)	C40—C41—C42	119.9 (3)
C12—C13—H13	119.5	C40—C41—H41	120.1
C14—C13—H13	119.5	C42—C41—H41	120.1
C15—C14—C13	119.8 (3)	C41—C42—C37	121.3 (3)
C15—C14—H14	120.1	C41—C42—H42	119.3
C13—C14—H14	120.1	C37—C42—H42	119.3
C16—C15—C14	119.7 (3)	N8—C43—C44	120.0 (2)
C16—C15—H15	120.2	N8—C43—H43	120.0
C14—C15—H15	120.2	C44—C43—H43	120.0
C15—C16—C17	120.4 (3)	C49—C44—C45	118.3 (2)
C15—C16—H16	119.8	C49—C44—C43	118.7 (2)
C17—C16—H16	119.8	C45—C44—C43	123.0 (2)
C12—C17—C16	120.4 (3)	O4—C45—C44	122.8 (2)
C12—C17—H17	119.8	O4—C45—C46	117.0 (2)
C16—C17—H17	119.8	C44—C45—C46	120.2 (2)
N4—C18—C19	119.6 (2)	C47—C46—C45	120.0 (3)
N4—C18—H18	120.2	C47—C46—H46	120.0
C19—C18—H18	120.2	C45—C46—H46	120.0
C24—C19—C20	118.3 (2)	C46—C47—C48	120.4 (3)
C24—C19—C18	118.4 (2)	C46—C47—H47	119.8
C20—C19—C18	123.3 (2)	C48—C47—H47	119.8
O2—C20—C21	117.6 (2)	C47—C48—C49	119.8 (3)
O2—C20—C19	122.5 (2)	C47—C48—H48	120.1
C21—C20—C19	119.9 (3)	C49—C48—H48	120.1
C22—C21—C20	120.4 (3)	C48—C49—C44	121.3 (3)
C22—C21—H21	119.8	C48—C49—H49	119.4
C20—C21—H21	119.8	C44—C49—H49	119.4
C21—C22—C23	120.7 (2)	C27—C50—H50A	109.5
C21—C22—H22	119.7	C27—C50—H50B	109.5
C23—C22—H22	119.7	H50A—C50—H50B	109.5
C24—C23—C22	118.9 (3)	C27—C50—H50C	109.5
C24—C23—H23	120.5	H50A—C50—H50C	109.5
C22—C23—H23	120.5	H50B—C50—H50C	109.5
			
C1—N1—N2—C2	0.4 (3)	C21—C22—C23—C24	−1.7 (4)
C3—N1—N2—C2	173.2 (2)	C22—C23—C24—C19	0.4 (4)
C2—N3—N4—C18	−164.4 (2)	C20—C19—C24—C23	1.1 (4)
C1—N3—N4—C18	22.8 (4)	C18—C19—C24—C23	−177.6 (3)
C26—N5—N6—C27	1.5 (3)	N6—N5—C26—N7	−1.1 (3)
C28—N5—N6—C27	172.5 (2)	C28—N5—C26—N7	−171.5 (2)
C27—N7—N8—C43	−174.7 (2)	N6—N5—C26—S2	177.96 (19)
C26—N7—N8—C43	−1.1 (4)	C28—N5—C26—S2	7.6 (4)
N2—N1—C1—N3	−1.6 (3)	C27—N7—C26—N5	0.4 (3)
C3—N1—C1—N3	−173.8 (2)	N8—N7—C26—N5	−173.7 (2)
N2—N1—C1—S1	175.61 (18)	C27—N7—C26—S2	−178.7 (2)
C3—N1—C1—S1	3.4 (4)	N8—N7—C26—S2	7.2 (4)
C2—N3—C1—N1	2.2 (2)	N5—N6—C27—N7	−1.2 (3)
N4—N3—C1—N1	175.4 (2)	N5—N6—C27—C50	179.4 (2)
C2—N3—C1—S1	−174.9 (2)	N8—N7—C27—N6	175.6 (2)
N4—N3—C1—S1	−1.7 (4)	C26—N7—C27—N6	0.5 (3)
N1—N2—C2—N3	1.1 (3)	N8—N7—C27—C50	−4.9 (4)
N1—N2—C2—C25	−177.3 (2)	C26—N7—C27—C50	−180.0 (2)
N4—N3—C2—N2	−176.5 (2)	C26—N5—C28—C37	93.8 (3)
C1—N3—C2—N2	−2.2 (3)	N6—N5—C28—C37	−76.2 (3)
N4—N3—C2—C25	1.9 (3)	C26—N5—C28—C29	−139.6 (2)
C1—N3—C2—C25	176.2 (2)	N6—N5—C28—C29	50.4 (3)
C1—N1—C3—C4	−133.4 (2)	N5—C28—C29—C30	64.9 (3)
N2—N1—C3—C4	54.6 (3)	C37—C28—C29—C30	−172.4 (2)
C1—N1—C3—C12	99.5 (3)	C28—C29—C30—O3	1.3 (4)
N2—N1—C3—C12	−72.4 (3)	C28—C29—C30—C31	−178.0 (2)
N1—C3—C4—C5	63.8 (3)	O3—C30—C31—C36	−3.7 (4)
C12—C3—C4—C5	−173.2 (2)	C29—C30—C31—C36	175.6 (2)
C3—C4—C5—O1	1.6 (3)	O3—C30—C31—C32	175.7 (3)
C3—C4—C5—C6	−178.5 (2)	C29—C30—C31—C32	−5.0 (4)
O1—C5—C6—C11	171.4 (2)	C36—C31—C32—C33	1.0 (4)
C4—C5—C6—C11	−8.4 (4)	C30—C31—C32—C33	−178.4 (2)
O1—C5—C6—C7	−7.6 (4)	C31—C32—C33—C34	0.0 (4)
C4—C5—C6—C7	172.6 (2)	C32—C33—C34—C35	−1.1 (4)
C11—C6—C7—C8	−2.4 (4)	C33—C34—C35—C36	1.2 (4)
C5—C6—C7—C8	176.6 (2)	C34—C35—C36—C31	−0.2 (4)
C6—C7—C8—C9	0.4 (4)	C32—C31—C36—C35	−0.9 (4)
C7—C8—C9—C10	1.7 (4)	C30—C31—C36—C35	178.5 (2)
C8—C9—C10—C11	−1.8 (4)	N5—C28—C37—C42	−93.6 (3)
C7—C6—C11—C10	2.3 (4)	C29—C28—C37—C42	142.0 (2)
C5—C6—C11—C10	−176.7 (2)	N5—C28—C37—C38	81.7 (3)
C9—C10—C11—C6	−0.2 (4)	C29—C28—C37—C38	−42.7 (3)
N1—C3—C12—C13	92.1 (3)	C42—C37—C38—C39	0.8 (4)
C4—C3—C12—C13	−32.0 (3)	C28—C37—C38—C39	−174.6 (2)
N1—C3—C12—C17	−82.6 (3)	C37—C38—C39—C40	−1.3 (4)
C4—C3—C12—C17	153.3 (2)	C38—C39—C40—C41	0.8 (4)
C17—C12—C13—C14	−0.1 (4)	C39—C40—C41—C42	0.2 (4)
C3—C12—C13—C14	−174.8 (2)	C40—C41—C42—C37	−0.7 (4)
C12—C13—C14—C15	−0.1 (4)	C38—C37—C42—C41	0.2 (4)
C13—C14—C15—C16	0.5 (4)	C28—C37—C42—C41	175.7 (2)
C14—C15—C16—C17	−0.7 (4)	N7—N8—C43—C44	177.3 (2)
C13—C12—C17—C16	0.0 (4)	N8—C43—C44—C49	−178.1 (3)
C3—C12—C17—C16	174.9 (2)	N8—C43—C44—C45	0.6 (4)
C15—C16—C17—C12	0.4 (4)	C49—C44—C45—O4	177.7 (3)
N3—N4—C18—C19	−179.3 (2)	C43—C44—C45—O4	−1.0 (4)
N4—C18—C19—C24	176.6 (2)	C49—C44—C45—C46	−1.6 (4)
N4—C18—C19—C20	−2.1 (4)	C43—C44—C45—C46	179.7 (2)
C24—C19—C20—O2	178.0 (2)	O4—C45—C46—C47	−178.1 (3)
C18—C19—C20—O2	−3.3 (4)	C44—C45—C46—C47	1.3 (4)
C24—C19—C20—C21	−1.4 (4)	C45—C46—C47—C48	0.1 (4)
C18—C19—C20—C21	177.3 (2)	C46—C47—C48—C49	−1.1 (4)
O2—C20—C21—C22	−179.3 (2)	C47—C48—C49—C44	0.7 (4)
C19—C20—C21—C22	0.2 (4)	C45—C44—C49—C48	0.7 (4)
C20—C21—C22—C23	1.4 (4)	C43—C44—C49—C48	179.4 (3)

### Hydrogen-bond geometry (Å, °)

**Table:**

*D*—H···*A*	*D*—H	H···*A*	*D*···*A*	*D*—H···*A*
O2—H2···N4	0.85 (3)	1.92 (3)	2.653 (3)	144 (3)
O4—H4···N8	0.95 (3)	1.80 (3)	2.658 (3)	149 (3)
C29—H29B···O1^i^	0.99	2.52	3.331 (3)	139 (2)
C38—H38···O1^i^	0.95	2.56	3.440 (3)	154 (2)

Symmetry codes: (i) *x*, *y*+1, *z*.
